# Camberwell Infirmary

**Published:** 1903-08-01

**Authors:** 


### CAMBERWELL INFIRMARY.

ON July 24th the new buildings of the Camberwell Infirmary, situated in Brunswick Square, were formally opened by the Chairman of the Board of Guardians, Mr. Alfred Foster.

A large company of people assembled to witness the proceedings. The resolution requesting the Chairman of the Board to declare the building open having been read, the Chaplain of the Infirmary, the Rev. E. Geard, offered a dedicatory prayer. Mr. Edwin T. Hall, the architect, then handed a key to the Chairman, with which to unlock the main door of the administrative building.

The Chairman said he accepted with sincere thanks the key with which he would shortly unlock the main door of the noble block of buildings, and would preserve the key as a memento both of the opening of the infirmary and of the time they had spent together during the construction of the building, and he thanked Mr. Hall in the name of the Board for his kind and valuable assistance.

The Chairman then went on to welcome the large gathering that had assembled, and invited them to inspect the building, so that they might learn what the Guardians had been doing during the last three years for the sick poor of that large parish. During the last eight or ten years the spirit of regard for suffering humanity, especially for the aged and very young, had been greatly stimulated, and the poor of the parish had been well attended to in Camberwell during that time. As an example, he believed the Camberwell Board was the first of the London Boards to adopt the system of scattered homes for the children. In 1873 the population of Camberwell was 111,302, while at the present day it was 259,339, and the inmates of the infirmary had increased in a corresponding ratio. With reference to the charge of extravagance, which was brought against them in common with all public bodies, he thought that after inspecting the building they would all be of opinion that the money had been expended wisely and well and not at all extravagantly.

The Chairman then said that on behalf of the Board of Guardians, and in the name of the Sovereign Healer of the whole world's pain, he declared the building open, and in doing so felt he was echoing the sentiments of all when he said that it was their hope and wish that the blessing of the Almighty Father might rest upon every effort put forth in this place to ameliorate pain, to restore health, and to comfort the last hours of those who must pass home to the world beyond.

Dr. Partridge, Chairman of the Infirmary Visiting Committee, said they had now a building that was capable of dealing with the work that was laid upon them. Fourteen years ago they had not one single trained nurse in the institution, now they had not a single nurse who was not either trained or in course of training, and yet the cost was no greater than it had been in the days of the "Sarah Gamps." Their nurses had obtained some of the most important posts in the Metropolis. They owed a very large debt of gratitude to their medical superintendent, Dr. Keats, and to the matron. The residents of Camberwell were a long way from any general hospital, and in consequence they received in their infirmary a large number of serious cases. Last year as many as 280 operations under chloroform were performed, and during the present year they had already had 220. Dr. Partridge concluded by saying that they were at the present time decidedly under-staffed, and made an energetic appeal to the Local Government Board to remedy this defect.

Dr. Macnamara, M.P., felicitated all concerned on the happy issue of their labours, and referring to the cost of the building, which amounted to about £200,000, said that no sum of money could have been better spent, but when they considered the fact that the whole payment had fallen upon the ratepayers of a comparatively poor parish, he thought the rate should be largely supplemented by a grant from the Imperial Exchequer.

A vote of thanks to the chairman was then proposed by the Mayor of Camberwell and seconded by Alderman Madge.

Mr. Hall, the architect, next gave a brief description of the building, in the course of which he mentioned that 600 men had been engaged in carrying out the work; and notwithstanding the extent of the operations there had not been a fatal accident, or any serious one. Mr. Hall also remarked that the building contained a mile and a half of drains, 16 miles of pipes, and 86 miles of electric wires. The building was then inspected by parties of 50, each under the guidance of a steward. The inspection occupied about an hour, and the visitors ended by partaking of tea in one of the wards, which had been beautifully decorated with flowers for the occasion.

